# Convalescent plasma therapy and mortality in COVID-19 patients admitted to the ICU: a prospective observational study

**DOI:** 10.1186/s13613-021-00867-9

**Published:** 2021-05-12

**Authors:** Stefan Hatzl, Florian Posch, Nazanin Sareban, Martin Stradner, Konrad Rosskopf, Alexander C. Reisinger, Philipp Eller, Michael Schörghuber, Wolfgang Toller, Zdenka Sloup, Florian Prüller, Katharina Gütl, Stefan Pilz, Alexander R. Rosenkranz, Hildegard T. Greinix, Robert Krause, Peter Schlenke, Gernot Schilcher

**Affiliations:** 1 Intensive Care Unit, Department of Internal Medicine, Medical University of Graz, Graz, Austria; 2 Division of Hematology, Department of Internal Medicine, Medical University of Graz, Graz, Austria; 3 Division of Oncology, Department of Internal Medicine, Medical University of Graz, Graz, Austria; 4 Department for Blood Group Serology and Transfusion Medicine, Medical University Graz, Graz, Austria; 5 Division of Rheumatology and Immunology, Department of Internal Medicine, Medical University of Graz, Graz, Austria; 6 Department of Anesthesiology and Intensive Care Medicine, Medical University Graz, Graz, Austria; 7 Clinical Institute for Medical and Chemical Laboratory Diagnostics, Medical University of Graz, Graz, Austria; 8 Division of Angiology, Department of Internal Medicine, Medical University of Graz, Graz, Austria; 9 Division of Endocrinology and Diabetology, Department of Internal Medicine, Medical University of Graz, Graz, Austria; 10 Division of Nephrology, Department of Internal Medicine, Medical University of Graz, Graz, Austria; 11 Section of Infectious Diseases and Tropical Medicine, Department of Internal Medicine, Medical University of Graz, Graz, Austria

**Keywords:** COVID-19, ICU, Intensive care, Convalescent plasma, Respiratory failure, Acute respiratory distress syndrome

## Abstract

**Background:**

This study aimed to quantify the potential survival benefit of convalescent plasma therapy (CVP) in critically ill patients with acute respiratory failure related to coronavirus disease-2019 (COVID-19).

**Methods:**

This is a single-center prospective observational cohort study in COVID-19 patients with acute respiratory failure. Immediately after intensive care unit (ICU) admission patients were allocated to CVP treatment following pre-specified criteria to rapidly identify those patients potentially susceptible for this treatment. A propensity score adjustment [inverse probability of treatment weighted (IPTW) analysis] was implemented to account rigorously for imbalances in prognostic variables between the treatment groups.

**Results:**

We included 120 patients of whom 48 received CVP. Thirty percent were female with a median age of 66 years [25th–75th percentile 54–75]. Eighty-eight percent of patients presented with severe acute respiratory failure as displayed by a median paO_2_/FiO_2_ ratio (Horowitz Index) of 92 [77–150]. All patients required any kind of ventilatory support with more than half of them (52%) receiving invasive ventilation. Thirty-day ICU overall survival (OS) was 69% in the CVP group and 54% in the non-CVP group (log-rank *p* = 0.049), respectively. After weighing the time-to-event data for the IPTW, the favorable association between CVP and OS became even stronger (log-rank *p* = 0.035). Moreover, an exploratory analysis showed an overall survival benefit of CVP therapy for patients with non-invasive ventilation (Hazard ratio 0.12 95% CI 0.03–0.57, *p* = 0.007)

**Conclusion:**

Administration of CVP in patients with acute respiratory failure related to COVID-19 is associated with improved ICU survival rates.

**Supplementary Information:**

The online version contains supplementary material available at 10.1186/s13613-021-00867-9.

## Background

In late fall of 2019 severe acute respiratory syndrome coronavirus 2 (SARS-CoV-2) emerged in Wuhan, China and has spread worldwide since then, infecting and killing millions of people. Coronavirus disease 2019 (COVID-19), is caused by SARS-CoV-2 and represents a major challenge for global health-care systems and especially intensive care units (ICU) [1]. COVID-19 mainly affects the respiratory system with some patients rapidly progressing to acute respiratory distress syndrome (ARDS) [2]. Since the outbreak, various treatment strategies have been investigated with only a minority of them providing convincing benefits [3–7].

Use of convalescent plasma (CVP) as “passive immunization” has a history going back to the 1890s and was the only method of treating some infectious diseases before the development of antimicrobial therapies [8]. The rationale behind this approach is to jump start the immune system with a passive antibody therapy in the combat against SARS-CoV-2 [9, 10]. Large observational studies demonstrated an adequate safety profile for SARS-CoV-2 CVP [11].

Despite the increasing number of clinical trials investigating CVP against SARS-CoV-2 infection, the effect of CVP on COVID-19 patient outcomes remain elusive so far including the timing and selection of patients for optimal treatment efficacy [12–17]. In this context, there is accumulating evidence that early administration of CVP and certain patient characteristics may be main determinants for treatment success of CVP in COVID-19 patients [13, 15, 17, 18]. Nevertheless, significant knowledge gaps remain on this issue, in particular, in terms of which specific patient groups are those with particular treatment success in response to CVP therapy for COVID-19.

Within this prospective case–control study, we aimed to evaluate the effects of CVP therapy on overall survival (OS) in critically ill patients primarily admitted to the ICU due to COVID-19 associated acute respiratory failure. Immediately after ICU admission, CVP treatment was administered in patients who were selected for this therapy based on pre-specified criteria that aimed to identify patients who did not yield anti-SARS-CoV-2 antibody conversion. Therefore, our main rationale was that progression of lung injury and death from COVID-19 might originate from the SARS-CoV-2 viral burden which cannot be cleared due to an insufficient host immune response characterized by the absence of anti-SARS-CoV-2 antibodies and that supplementation of those antibodies by CVP should lead to clinical benefit.

## Methods

### Study cohort

**Fig. 1:**
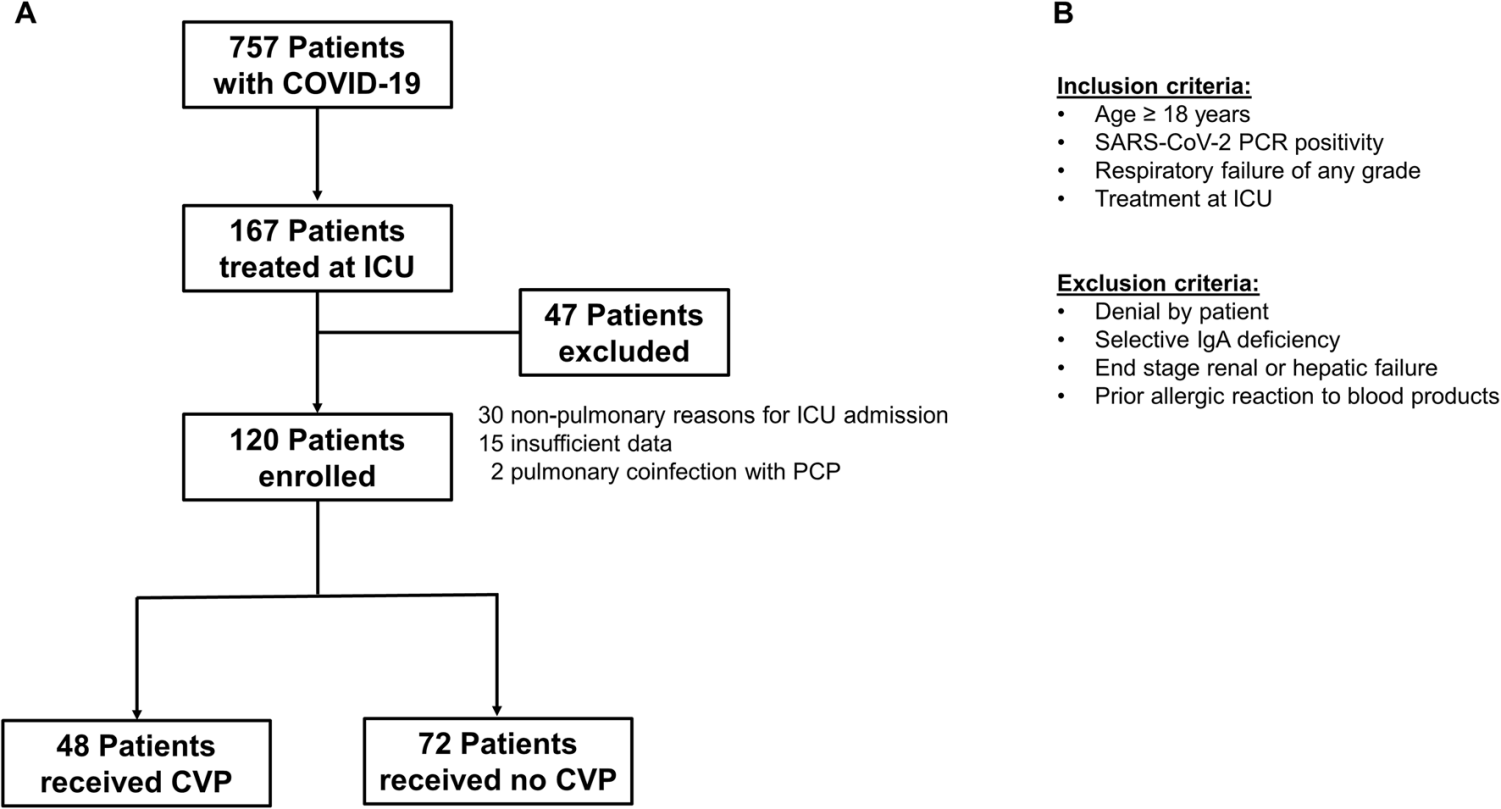
Full trial protocol and flow diagram. **a** Recruitment tree: 757 patients were initially considered for the study. 167 were treated at intensive care units (ICU). 47 patients were excluded—thereof 30 patients were excluded because of other than pulmonary indications for ICU admission. SARS-CoV-2 positivity was an incidental finding without any radiologic evidence of pneumonia. 15 were treated due to non-ST or ST-elevation myocardial infarction (NSTEMI/STEMI); 11 received post-operative intensive care treatment—3 patients were incidentally tested positive for SARS-CoV-2 due to ischemic or hemorrhagic stroke; 1 patient was incidentally tested positive for SARS-CoV-2 after cerebral bleeding within the work-up for organ donation, 15 patients were excluded due to insufficient follow-up data and 2 patients were excluded, because they had pulmonary co-infection with pneumocystis jirovecii (PJP). **b** Inclusion and exclusion criteria of the study. SARS-CoV-2 severe acute respiratory syndrome corona virus 2; PCR: polymerase chain reaction; ICU: intensive care unit; IgA: immunoglobulin A

For this prospective exposed–non-exposed cohort study (where exposed are the patients who received CVP and non-exposed those who did not), we considered all 757 consecutive SARS-CoV-2 polymerase chain reaction (SARS-CoV-2 PCR) positive patients who were referred for the treatment of COVID-19 at the Department of Internal Medicine, Medical University of Graz, Austria, between March 13th, 2020 and Jan 5th, 2021. All patient data was prospectively and uniformly collected according to a guidance which was locally released in March 2020 as a standard operating procedure (SOP) at our department. Laboratory and radiology data were extracted from our in-house electronic healthcare database system as described previously [19]. Final analysis (Fig. 1a) was performed after exclusion of patients who had not been admitted to the ICU (*n* = 586) or had received prior CVP therapy (*n* = 4), patients with non-pulmonary reason for ICU admission (*n* = 30), patients with insufficient follow-up data (*n* = 15) and patients with pulmonary P. jirovecii (PJP) co-infection (*n* = 2). In- and exclusion criteria are tabulated in Fig. 1b. The research project was approved by the local institutional review board (EK-Nr.: 32-475 ex 19/20).

### Indication and rational for CVP administration

Indication and administration of CVP has been informed by a standard operating procedure (SOP) which was published in Mach 2020 as local guidance for the Department of Internal Medicine, Medical University of Graz, Austria. Allocation of CVP therapy was made by an attending ICU or ID (infectious disease) physician. CVP therapy was restricted to COVID-19 patients admitted to ICU due to acute respiratory failure with acquired (after chemotherapy, under immunosuppression) or inborn immune deficiency (for detailed information about the immunosuppression see Additional file 1) or patients with negative SARS-CoV-2 antibody status at ICU admission.

### Evaluation of anti-SARS-CoV-2 antibodies in patients

To evaluate the serological status of patients admitted to ICU, total antibodies (including IgG and IgM) against SARS-CoV-2 nucleocapsid were measured using the Roche Elecsys® Anti-SARS-CoV-2 immunoassay (Roche Diagnostics, Rotkreuz, Switzerland) on a Cobas e801 analyzer platform (specificity > 99%) as described previously [20]. A cut-off index > 1 was regarded as positive.

### CVP collection and donor evaluation

COVID-19 convalescent apheresis plasma was collected at the Department of Blood Serology and Transfusion Medicine, Medical University Graz, Austria, between April 10th and November 11th 2020. Only clinical fully recovered COVID-19 patients donated plasma according to the recommendations of the European Commission and standard operating procedures of our department after written informed consent. To ensure therapeutic potential of CVP a minimum of 30 AU/ml neutralizing anti-SARS-CoV-2 S1/S2 IgG antibodies (DiaSorin®, Vienna, Austria) were determined as a release criterion of CVP. CVP was produced according to good manufacturing practice (GMP) guidelines and additional pathogen inactivation was performed using INTERCEPT Blood System (Cerus E.V., Amersfoort, The Netherlands) which is described in more detail in Additional file 2.

### Statistical analysis

All statistical analyses were performed using Stata (Windows version 15.1; Stata Corp., Houston, TX, USA). The distribution of baseline variables between the CVP and non-CVP group was evaluated using rank-sum tests, χ^2^ tests, and Fisher’s exact tests, as appropriate. The magnitude of these differences was quantified with standardized mean differences (SMDs), and SMDs ≥ 0.30 were considered indicative of a potentially relevant magnitude of difference. We obtained the propensity score *e* from a multivariable logistic regression model with treatment group as the outcome variable. For this model, we pre-specified a fixed number of 10 predictor variables, in order not to have less than 5 events per predictor variable. In detail, the model was developed by initially including all variables with a *p* for difference between the two groups of ≤ 0.10 and SMD ≥ 0.30. The following variables were excluded from the model development process: (1) mSOFA (modified sequential organ failure assessment) score and acute respiratory failure grading (inspired by acute ARDS Berlin 2012 classification) (due to strong collinearity with the Horowitz index); (2) white blood count (due to strong collinearity with the neutrophil count); and (3) the SARS-CoV-2 antibody status variable (due to the so-called perfect prediction problem), and the immunosuppression variable (due to the introduction of model instability by this variable). This model was then reduced with simple backward elimination to yield the pre-specified 10 predictor variable model. The propensity score *e* was then transformed into an inverse-probability-of-treatment-weight (IPTW) according to the average treatment effect principle, i.e., $=\frac{\text{CVP}}{e}+\frac{1-\text{CVP}}{1-e}$, where CVP denotes the treatment assignment (0 = non-CVP group, 1 = CVP group) [21]. Following best practice recommendations for balance diagnostics, we then re-estimated SMDs and *p* values for difference between the two treatment groups with the IPTW-weighted data [22, 23]. The primary outcome of the study, 3-month overall survival, was defined as the time interval from ICU admission to death-from-any-cause or the censoring date when being still alive 3 months after ICU admission. The 30-day ICU survival was defined as co-primary outcome to get further insights in the early ICU mortality of COVID-19 patients. Survival time was inflated by 1 day in patients who died at the day of ICU admission (*n* = 5). OS was computed with Kaplan–Meier estimators, and compared between the two treatment groups using log-rank tests. Uni- and multivariable modelling of mortality hazards was performed with Cox regression models. No evidence for a violation of the proportional hazards assumption according to treatment assignment was observed. These time-to-event analyses were then re-performed with IPTW weighting to control for selection bias. Sensitivity analyses included a trimmed IPTW (i.e., excluding patients with an IPTW ≤ the 1st and ≥ the 99th percentile of its distribution), and multivariable adjustment for variables that had high SMDs after IPTW weighting. In an exploratory hypothesis generating analysis we fitted interactions between treatment assignment and pre-specified subgroups (age ≤/> 65 years, number of comorbidities ≤/> 3, mSOFA ≤/> 5 points, paO_2_/FiO_2_ ≤/> 100, and Ferritin ≤/> 1500 mg/dl) to gauge whether the “effect” of CVP on OS may be modified by these clinical co-variables. The full dataset and the main analysis code are available on request by the first author.

## Results

### ICU cohort and CVP administration

One hundred twenty patients were admitted to the ICU due to COVID-19 associated acute respiratory failure, with 48 (40%) patients receiving CVP immediately after ICU admission according to our local standard (Table 1, Fig. 1). At the time of ICU admission, the median age of the cohort was 66 years [25th–75th percentile: 54–75], and 36 (30%) patients were female. The study population had a median of three comorbidities [1–4] and a median time from COVID-19 symptom onset to ICU admission of 4.0 days [1–6]. All patients included in this study showed an oxygen saturation of less than 88% while breathing ambient air prior to ICU admission. Most patients presented with moderate to severe respiratory failure as displayed by a median paO_2_/FiO_2_ ratio (Horowitz Index) of 92 [77–150]. The median anti-SARS-CoV-2 antibody concentration in CVP was 79.2 AU/ml [46.6–99.7], with a median dose of 600 ml CVP [600–600] administered in two applications (400 ml day 1, 200 ml day 2) or three subsequent daily doses of 200 ml depending on fluid tolerance of the patients. During a median follow-up of 1.9 months, we observed 52 deaths, corresponding to 1-, 2-, and 3-month OS estimates of 60% (95% CI 51–68), 54% (45–63), and 54% (45–63), respectively, in the complete cohort comprising 120 patients. (Additional file 3). Importantly, no unexpected or serious adverse events related to CVP administration were observed during the study period thereby encouraging the previously published outstanding safety profile of this therapy additionally regarding usage in ICUs.

**Table 1:** Baseline characteristics of the study population—distribution at ICU admission

Variable	Overall (*n* = 120)	CVP (*n* = 48)	Non-CVP (*n* = 72)	*p*	*p*_*IPTW*_
Demographic variables					
Age (years)	66 [54–75]	61 [53–72]	69 [55–76]	0.044	0.724
Female Gender	36 (30%)	12 (25%)	24 (33%)	0.417	0.277
BMI (kg/m^2^)	27.7 [24.5–32.5]	29.8 [25.0–34.4]	27.1 [23.9–31.3]	0.058	0.558
“First COVID-19 wave”	28 (23%)	3 (6%)	25 (35%)	< 0.001	0.194
Coexisting conditions					
Number of coexisting conditions	3 [1–4]	3 [2–5]	2 [1–4]	0.019	0.905
Hypertension	94 (78%)	43 (90%)	51 (70%)	0.022	0.838
Diabetes	46 (38%)	23 (48%)	23 (33%)	0.088	0.825
Atrial fibrillation	24 (20%)	15 (31%)	9(13%)	0.019	0.163
Coronary heart disease^$^	31 (26%)	14 (29%)	17 (24%)	0.520	0.457
Congestive heart failure	30 (25%)	17 (24%)	13 (27%)	0.672	0.738
Peripheral arterial disease	23 (19%)	15 (21%)	8 (17%)	0.641	0.195
Thromboembolic disease	18 (15%)	10 (21%)	8 (11%)	0.132	0.680
Chronic renal failure	35 (24%)	18 (38%)	17 (24%)	0.107	0.994
Dialysis	9 (8%)	3 (6%)	6 (8%)	0.740	0.104
COPD	16 (13%)	10 (14%)	6 (13%)	1.000	0.742
Asthma	13 (10%)	8 (17%)	5 (7%)	0.130	0.498
Prior cancer in complete remission	13 (10%)	8 (17%)	5 (7%)	0.507	0.226
Active malignancy	9 (8%)	5 (10%)	4 (6%)	0.105	0.228
Dementia	4 (3%)	2 (4%)	2 (3%)	1.000	0.947
Prior organ transplantation	5 (4%)	5 (10%)	0 (0%)	0.009	N/E
Immunosuppression^†^	18 (15%)	15 (30%)	3 (4%)	< 0.001	0.009
ICU risk stratification					
mSOFA (points)	5 [4–7]	6 [4–8]	4 [3–5]	< 0.001	0.204
paO_2_/FiO_2_	92 [77–150]	81 [71–96]	113 [83–186]	< 0.001	0.198
PEEP (mmHg)—maximum	11 [9–12]	11 [9–13]	10 [9–12]	0.157	0.623
Acute respiratory failure grade (inspired by ARDS *Berlin 2012*-classification)	/	/	/	< 0.001	0.140
Severe	76 (63%)	42 (88%)	34 (47%)	/	/
Moderate	30 (25%)	6 (13%)	24 (33%)	/	/
Mild	14 (12%)	0 (0%)	14 (19%)	/	/
Ventilation (maximum invasivity)	/	/	/	0.400	0.364
Intubated	55 (46%)	25 (52%)	30 (42%)	/	/
vvECMO	7 (6%)	3 (6%)	4 (6%)	/	/
NIV	47 (39%)	18 (38%)	29 (40%)	/	/
HFNC	11 (9%)	2 (4%)	9 (13%)	/	/
Any invasive ventilation	62 (52%)	28 (58%)	34 (47%)	0.233	0.130
CVP characteristics					
Length of symptoms before first transfusion (days)	/	4 [1–10]	/	/	
Antibody concentration (AU/ml)	/	79.2 [46.6–99.7]	/	/	
Administered dose of CVP (ml)	/	600 [600–600]	/	/	
Laboratory values					
Lactate (mmol/l)	2.7 [1.6–3.8]	2.9 [2.3–4.1]	1.9 [1.2–3.1]	0.003	0.646
IL-6 (pg/ml)*	125 [65–245]	171 [85–334]	104 [40–204]	0.017	0.680
CRP (mg/l)	110 [63–187]	145 [104–200]	96 [42–169]	< 0.001	0.157
Ferritin (ng/ml)*	1240 [523–2090]	1940 [799–2829]	910 [456–1479]	< 0.001	0.028
hs-TnT (pg/ml)*	18 [9–31]	18 [8–26]	19 [11–54]	0.200	0.263
d-Dimer (mg/l)*	2.3 [1.1–4.8]	2.5 [1.4–5.4]	2.0 [0.9–4.7]	0.172	0.484
SARS-CoV-2-Antibody positivity at ICU admission	49 (49%)	3 (7%)	46 (85%)	< 0.001	0.001
Blood counts					
Leukocytes [G/l]	8.3 [5.1–11.4]	6.0 [4–1–10.1]	8.7 [6.3–12.3]	0.007	0.020
Neutrophiles [G/l]	6.5 [2.4–9.1]	4.6 [3.0–7.6]	7.2 [4.0–9.4]	0.005	0.042
Lymphocytes [G/l]	0.7 [0.5–1.2]	0.7 [0.4–1.1]	0.7 [0.5–1.2]	0.226	0.694
Thrombocytes [G/l]	174 [129–279]	179 [135–292]	177 [127–236]	0.415	0.006
Specific medication					
Glucocorticoids^§^	120 (100%)	48 (100%)	72 (100%)	1.000	N/A
Remdesivir	19 (17%)	8 (17%)	11 (15%)	0.796	0.215
Hydroxychloroquin	15 (13%)	6 (13%)	9 (13%)	1.000	
Tocilizumab	6 (5%)	4 (8%)	2 (3%)	0.403	N/A

Data are reported as medians [25th‐75th percentile] or as absolute counts (%)

*p* denotes *p* values before ITPW weighting, *p*_IPTW_ denotes *p* values after IPTW adjustment

*p*‐values are either from rank‐sum tests, χ^2^‐tests, or Fisher's exact tests, as appropriate

CVP: convalescent plasma; BMI: body mass index; ICU: intensive care unit; mSOFA: modified sequential organ failure assessment; PEEP: positive end expiratory pressure; vvECMO: veno-venous extracorporal membrane oxygenation; IV: invasive ventilation; NIV: non-invasive ventilation; HFNC: high flow nasal cannula; IL-6: interleukin 6; CRP: C-reactive protein; hs-TnT: high sensitive troponin T, SARS-CoV2: severe acute respiratory syndrome corona virus 2

^$^Documented coronary heart disease either by specific coronary imaging or coronary angiography

^†^Comprises immunosuppressive medication (low dose of glucocorticoids are excluded) as well as diseases with severe immunosuppression

^§^Glucocorticoids included low-dose dexamethasone or equivalent doses of other glucocorticoids

^*^Reports variables with missing values: IL-6—118 patients fully observed (2% missing), Ferritin—118 patients fully observed (2% missing), hs-TnT—119 patients fully observed (1% missing), d-Dimer—119 patients fully observed (1% missing), SARS-CoV-2-antibody positivity at ICU admission—100 patients fully observed (17% missing)

### Overall survival according to CVP administration—crude analysis

In a first step we evaluated our CVP allocation strategy by anti-SARS-CoV-2 anybody status at ICU admission and/or history of inborn or acquired immunosuppression. Therefore, we performed an unadjusted “crude” analysis of our data.

**Fig. 2:**
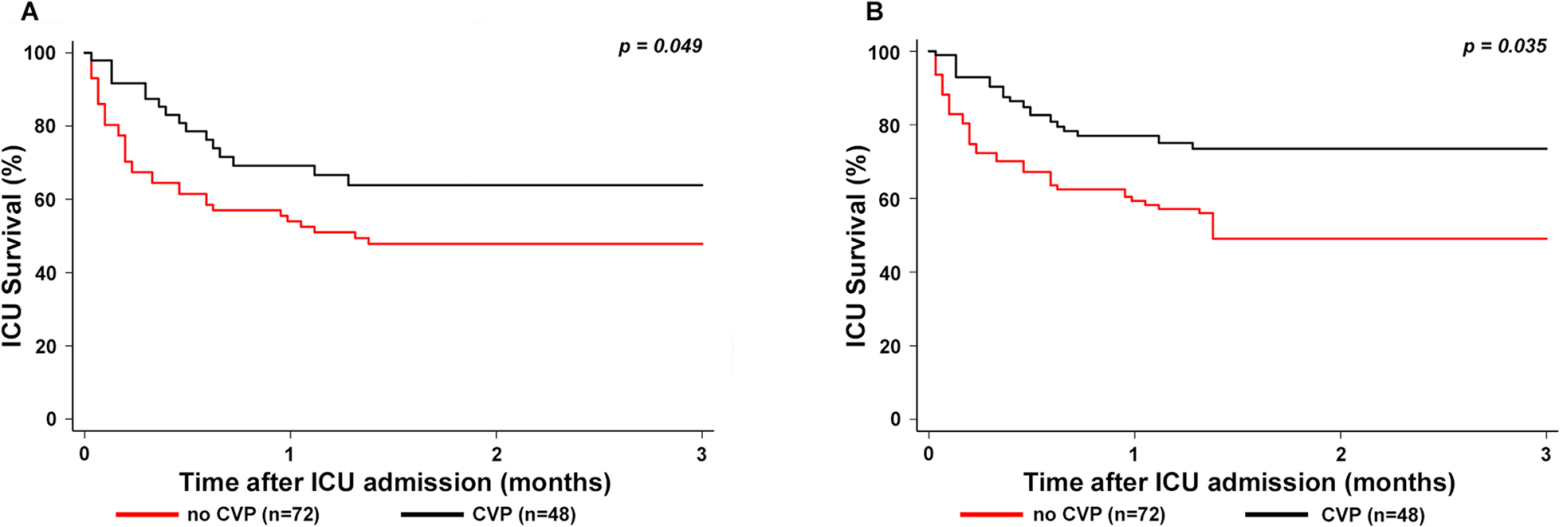
Survival function according to CVP status. **a** Unadjusted analysis—median overall survival estimates were not reached in the convalescent plasma (CVP) group and 1.3 month in the non-convalescent plasma (non-CVP) group; 30-day survival estimates were 69% in the CVP group and 54% in the non-CVP group. Statistical significance calculated using the log rank test. A *p* value < 0.05 was considered statistically significant. **b** IPTW-weighted analysis—median overall survival estimates were not reached in the convalescent plasma (CVP) group and 1.3 month in the non-convalescent plasma (non-CVP) group; 30-day survival estimates were 77% in the CVP group and 59% in the non-CVP group. Statistical significance calculated using the log rank test. A *p *value < 0.05 was considered statistically significant

In crude analysis, patients given CVP experienced a more favorable 3-month OS than patients who did not receive CVP. In detail, after 1.3 months 50% of patients deceased in the non-CVP group, whereas patients allocated to CVP treatment did not reach this threshold (log-rank *p* = 0.049, Fig. 2a). To get further insights into the early ICU mortality, we calculated the 30-day OS which estimates 69% in the CVP group, and 54% in the non-CVP group. In univariable Cox regression, this corresponded to a Hazard Ratio (HR) of 0.56 (95% CI 0.31–1.01, *p* = 0.054) by CVP therapy.

### Development of a propensity score

On average, patients who received CVP therapy had baseline covariates consistent with more severe critical illness (Table 1, Additional file 4). Specifically, as indicated by standardized mean differences (SMD), patients in the CVP therapy group had a higher number of comorbidities, a higher modified sequential organ failure assessment (mSOFA) score, a lower paO_2_/FiO_2_ ratio, more severe acute respiratory failure grade (inspired by ARDS *Berlin 2012*-classification), and higher levels of adverse predicting laboratory parameters such as lactate, interleukin-6, and C-reactive protein. Otherwise, patients in the CVP group were significantly younger and less likely to be treated in the “first wave” of COVID-19 (March–May 2020), and (consistent with the local guidance for CVP administration) had a higher prevalence of immunosuppression and negativity for SARS-CoV-2 antibodies (Table 1). These imbalances are consistent with the non-random assignment to CVP by treating physicians, likely underestimating potential beneficial effects of CVP therapy (i.e., a “conservative” bias). To control for this potential bias, we predicted a propensity score based on a 10-variable multivariable logistic regression model (Table 2). The propensity score covered the whole probability range (Additional file 5-Panel A) and was then transformed into the IPTW (Additional file 5-Panel B). Re-weighting of the data strongly reduced many but not all differences in baseline covariates between the two treatment groups (Table 1, Additional file 6), which we considered to be indicative of adequate balance that requires additional multivariable adjustment for selected variables (immunosuppression, SARS-CoV-2 antibody positivity, paO_2_/FiO_2_ ratio, and “first wave” of COVID-19) within a sensitivity analysis.

**Table 2:** Propensity score model for treatment group assignment

Variable	Multivariable odds ratio (OR)	95% CI	*p*
Demographic variables			
Age (per 5 years increase)	0.67	0.51–0.89	0.006
“Firstwave” of COVID-19 (per 30-day increase)	1.80	1.31–2.48	< 0.0001
Number of comorbidities (per 1 condition increase)	1.98	1.27–3.07	0.002
Chronic renal failure	0.16	0.02–1.04	0.05
Active malignancy	8.03	0.26–250.22	0.235
paO_2_/FiO_2_ (per 10 units increase)	0.72	0.60–0.87	0.001
Lactate (per 1 mmol/l increase)	0.77	0.60–0.99	0.038
CRP (per 50 mg/dl increase)	1.44	0.98–2.12	0.063
Ferritin (per 1000 ng/ml increase)	1.25	0.95–1.64	0.107
Absolute neutrophil count (per 1 G/increase)	0.76	0.63–0.92	0.004

### Overall survival according to CVP administration—IPTW analysis

We then sought to reduce non-random assignment effects within our study population. Therefore, we weighted the time-to-event data for the IPTW and could demonstrate that the favorable association between CVP and OS became even stronger (IPTW-weighted log-rank *p* = 0.035, Fig. 2b). We calculated the 30-day OS after weighting the time-to event data which were 77% in the CVP group, and 59% in the non-CVP group. This corresponded to an estimated 2.3-fold lower risk of death (IPTW-adjusted HR = 0.44, 0.21–0.95, *p* = 0.035) after CVP therapy. This association prevailed after multivariable adjustment for covariates with clear prognostic relevance (Additional file 4) and/or an insufficiently reduced imbalance between the two treatment groups (as reported in Table 1), and/or variables that by local guidance led to a recommendation for CVP administration (immunosuppression, negativity for SARS-CoV-2 antibodies, Table 3). Here, besides CVP, the strongest multivariable independent predictor for a more favorable OS was positivity for SARS-CoV-2 antibodies at ICU admission. Even though stringent inclusion criteria were applied for CVP allocation three patients did not receive CVP despite a negative anti-SARS-CoV-2 antibody status (Table 1) allowing this analysis.

**Table 3:** Multivariable Cox regression of overall survival according to convalescent plasma therapy in the overall cohort (*n* = 118)

Multivariable model	Variable	Multivariable hazard ratio	95% CI	*p*
#1 (*n* = 118)	CVP	0.39	0.19–0.81	0.011
	paO_2_/FiO_2_ (per 10 units increase)	0.87	0.80–0.95	0.002
#2 (*n* = 118)	CVP	0.39	0.18–0.84	0.016
	mSOFA (per 1 point increase)	1.26	1.08–1.46	0.003
#3 (*n* = 118)	CVP	0.29	0.13–0.66	0.003
	Positivity for SARS-COV-2 antibodies	0.46	0.22–0.95	0.036
#4 (*n* = 118)	CVP	0.38	0.17–0.85	0.018
	Immunosuppression	1.75	0.65–4.68	0.265
#6 (*n* = 98)	CVP	0.21	0.09–0.47	< 0.0001
	Positivity for anti-SARS-COV-2 antibodies	0.39	0.18–0.85	0.018
	mSOFA (per 1 point increase)	1.30	1.10–1.54	0.003
	Immunosuppression	1.40	0.51–3.81	0.515

Two patients were excluded because of missing variables. Model#1 is CVP adjusted for paO_2_/FiO_2_, Model#2 is CVP adjusted for mSOFA-modified sequential organ failure assessment, Model#3 is CVP adjusted for anti-SARS-CoV-2 antibodies positivity at time of ICU admission, Model#4 is CVP adjusted for immunosuppression at time of ICU admission, Model#5 is CVP adjusted for anti-SARS-CoV-2 antibodies positivity at time of ICU admission, mSOFA and immunosuppression In these analyses, the association between CVP and a higher risk of death prevailed after multivariable adjustment

### Impact of CVP antibody concentration and time to infusion

In those 48 patients receiving CVP therapy, higher antibody concentrations did not emerge as a predictor of more favorable survival outcome (HR per 50 AU/ml increase = 1.07, 95% CI 0.62–1.85, *p* = 0.815). Similarly, time from diagnosis of COVID-19 to CVP infusion was not associated with survival at 3 months (HR per 5-day increase = 0.70, 95% CI 0.41–1.19, *p* = 0.187).

### Exploratory analysis of predictive markers

**Fig. 3:**
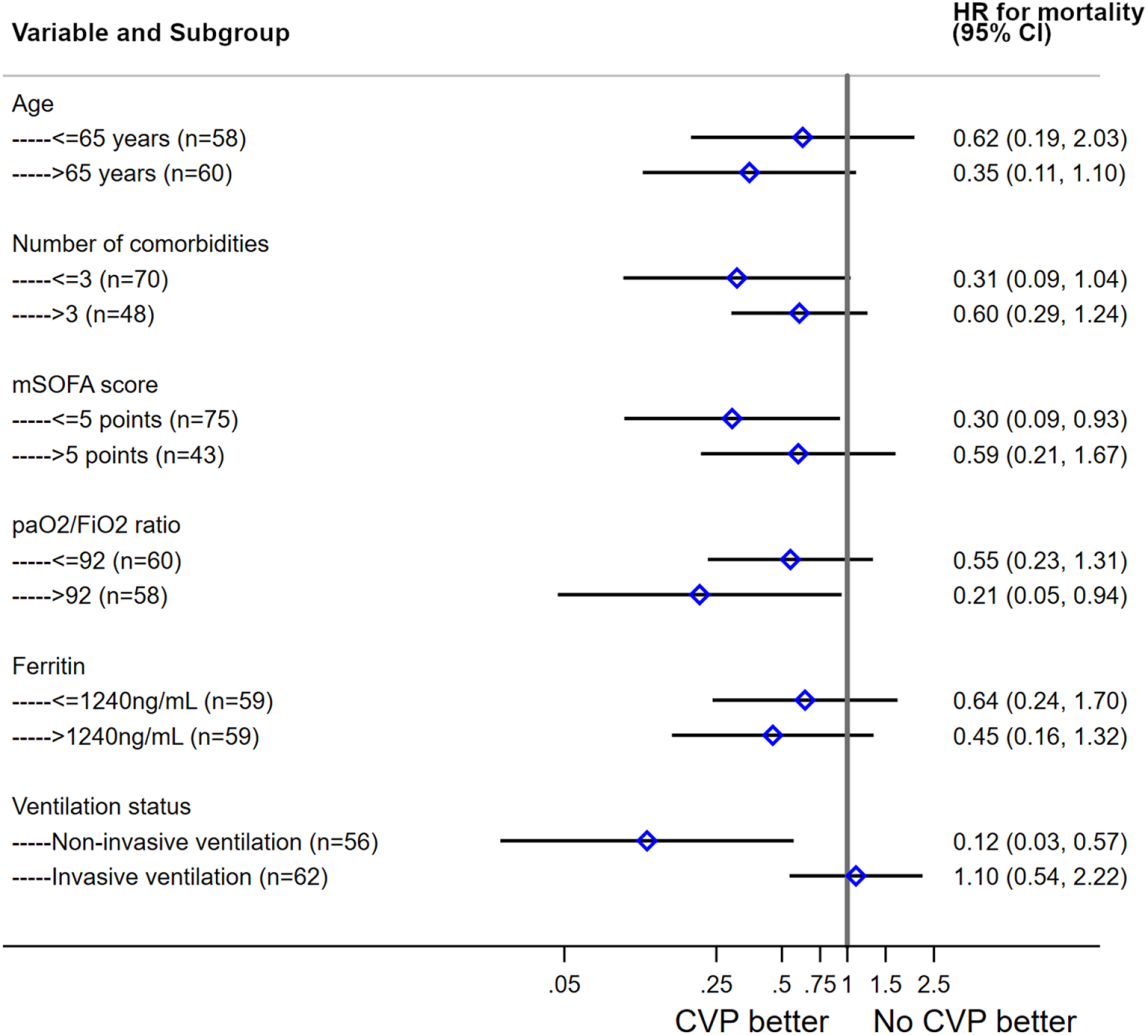
Subgroup analysis—forest plot of the relative association of CVP with overall survival according to selected clinical co-variables. Hollow-blue diamonds represent the subgroup hazard ratio, and the associated bars the 95% confidence interval. The black vertical line represents the “line of unity”, at which patients who were and were not treated with CVP have similar hazards of overall mortality. Regression results were obtained by fitting an interaction between the CVP treatment and the respective subgroup variable

In subgroup analyses using IPTW-adjusted Cox models, the association between CVP and favorable survival outcome was consistent across pre-specified subgroups defined by age (interaction *p* = 0.496), comorbidities (*p* = 0.800), mSOFA score (*p* = 0.600), and ferritin levels (*p* = 0.570), but not across the paO_2_/FiO_2_ ratio (Fig. 3). Here, CVP appeared to interact with the paO_2_/FiO_2_ ratio (interaction *p* = 0.033), which prompted us to explore the potential modifying “effect” of invasive ventilation on the association between CVP and survival. In this hypothesis generating, post-hoc analysis, the potential positive effect of CVP appeared to be confined to patients on non-invasive ventilation (Fig. 3). In detail, IPTW-adjusted HRs for CVP were 0.12 (95% CI 0.03–0.57, *p* = 0.007) in those with non-invasive ventilatory support, and 1.10 (0.54–2.22, *p* = 0.798) in those with invasive ventilatory support, respectively (interaction *p* = 0.011).

## Discussion

Within this prospective cohort study CVP therapy was associated with improved OS in severe to life-threatening diseased COVID-19 patients with absence of anti-SARS-CoV-2 antibody seroconversion at time of ICU admission. The survival benefit of CVP therapy was even evident in crude analyses although patients receiving CVP had an increased risk profile compared to those not selected for this treatment. In addition, we were able to highlight that absence of anti-SARS-CoV-2 antibodies in patients at time of ICU admission is one of the strongest multivariable predictors of worse ICU outcomes in our cohort, strengthening the rational of our in-house defined protocol to treat those patients with CVP.

The presence of anti-SARS-CoV-2 antibodies indicates an active targeted immune response with the potential to limit viral spread. Patients unable to mount a timely antibody response may be in disadvantage as they rely merely on the anti-viral capacities of innate immune cells and T cells [24–26]. Our herein provided beneficial outcome results for patients receiving CVP are consistent with aggregated data from non-randomized and randomized clinical trials showing beneficial outcomes with regard to CVP [13, 15, 17, 18]. Similarly, those studies focused on special characterized cohorts to tailor a personalized treatment for critically ill COVID-19 patients. Our patients received CVP early after onset of symptoms (median 4 days; 25th–75th percentile: 1–10). This fact agrees with clinical trials showing favourable outcomes due to CVP [13, 15, 17, 18] but is in contrast to those who failed underlining the need for more data on this topic as provided by our investigation [12, 14, 16, 27, 28]. In addition, two large clinical trials could highlight the importance of high anti-SARS-CoV-2 antibody concentrations of CVP as well as earlier administration of CVP in the course of the disease [17, 18]. Interestingly, neither higher antibody concentration of CVP nor time from COVID-19 diagnosis to CVP infusion were associated with better CVP effects. This fact might be biased by case allocation driven by sero-positivity of patients which vanishes the effect of higher antibody concentration as well as early severity of COVID-19 which vanishes the effect of time to infusion.

We could demonstrate that ICU treatment during the first wave of COVID-19 was an independent negative prognostic marker of ICU outcomes (Table 2). Similar findings of declining ICU mortality during the pandemic waves have been published by other groups and might reflect learning processes and deeper knowledge of disease mechanisms as well as an optimization of ventilation concepts and other ICU treatment strategies [29, 30]. However, any potential time dependent bias of CVP administration has been eliminated by our statistical calculations using IPTW weighted analysis.

In an exploratory analysis we could demonstrate that patients with non-invasive ventilation benefit most from CVP therapy. One might speculate that more severe acute respiratory failure requiring invasive ventilation has advanced from an inflammatory to a more proliferative phase. While proliferative alveolar damage is frequently found upon autopsy of COVID-19 cases, anti-viral therapy such as CVP might not be able to improve the course of disease at that stage [31]. Therefore, our findings suggest that those patients receiving non-invasive ventilation should be rapidly evaluated for CVP treatment after ICU admission to reduce the risk of intubation and improve their OS.

Limitations of our study are its observational nature, the imbalances of the patient characteristic regarding their specific risk profiles and the relatively long timeframe of study inclusion, which might demonstrate a bias as discussed above. However, we can provide high data quality by prospective collection and inclusion in our registry and we used very stringent inclusion criteria to reduce any bias. To account this non-random and time depended assignment effects of our study population we performed a propensity score adjusted analysis which has been described as a potent statistical method in the field of critical care [32]. By this analysis we were able to balance the risk factors between both groups and generate a sufficient pseudo-population which further strengthened our finding of beneficial effects of CVP on ICU mortality.

To the best of our knowledge, our investigation is the first prospective cohort study evaluating CVP therapy on OS in a severely ill and well characterized ICU cohort. Importantly, the baseline as well as ICU specific characteristics of our study population were demonstrate the sickest subgroup of a large recently published European ICU cohort comprising more than 4000 patients [30]. This supports the relevance and generalizability of our findings to all disciplines in charge of COVID-19 patient care.

## Conclusion

In this observational study comprising 120 critically ill patients and high proportion of immunocompromised patients with PCR confirmed COVID-19 and associated acute respiratory failure admitted to our ICUs, we were able to demonstrate that CVP treatment is able to improve ICU outcomes especially in patients with absence of anti-SARS-CoV-2 antibodies at time of ICU admission. We could further strengthen this finding using propensity scores to balance the population for known and unknown risk factors. In summary, we report the utility of CVP in a “real-world” ICU-cohort of critically ill COVID-19 patients highlighting the potential relevance of our finding to the field of intensive care medicine.

## Supplementary Information

**Additional file 1: Table S1.** Tabulation of immunocompromised patients. R-CHOP21—combination chemotherapy consisting of rituximab, cyclophosphamide, vincristine, prednisolone given in 21-day cycles; FOLFOX combination chemotherapy consisting of folic acid, 5-fluorouracil and oxaliplatin; DA 7 + 3—combination chemotherapy consisting of cytarabine given continuously for 7 days and daunorubicin given on 3 consecutive days.

**Additional file 2.** Supplementary Materials and Methods.

**Additional file 3: Figure S1.** Overall survival of the whole cohort (*n* = 120). Median follow-up of the whole cohort was 1.9 months (with follow-up truncated at 3 months), we totally observed 52 deaths which corresponds to 1-, 2-, and 3-month OS estimates of 60% (95% CI 51–68), 54% (45–63), and 54% (45–63). CI: confidential interval.

**Additional file 4: Table S2.** Univariable predictors of survival in the cohort (*n* = 120). BMI: body mass index; first wave: depicts patients treated during the first wave of COVID-19 in Austria between March 2020 and August 2020; mSOFA: modified sequential organ failure assessment, maxPEEP: depicts the maximum applied PEEP to achieve optimal ventilation; IL-6: interleukin 6; CRP-C-reactive protein; hs-TNT: high sensitive troponin T

**Additional file 5: Figure S2.** Histograms of the Propensity Score and the IPTW. (A) The propensity score can range from 0 to 1. Multiply by 100 to obtain probabilities (in percent) of having received CVP. (B) The IPTW was defined as the inverse of the probability of receiving the treatment that the patient received (i.e., the so-called “average treatment effect on the treated

**Additional file 6: Figure S3.** Standardized mean difference (SMD) plot. Blue squares denote Standardized mean differences (SMDs) in before weighting the inverse of the probability of treatment weight (IPTW). Yellow diamonds denote the SMDs after weighting the IPTW.

## Data Availability

Full patient data set and STATA analysis code are available on request by SH.
