# Supplementary material for: Convalescent plasma therapy and mortality in COVID-19 patients admitted to the ICU: a prospective observational study
Source: Ann Intensive Care. 2021 May 12;11:73. doi: 10.1186/s13613-021-00867-9 (PMC8114671; doi:10.1186/s13613-021-00867-9)
Supplement: Supplementary file 1 — Additional file 1: Table S1. Tabulation of immunocompromised patients. R-CHOP21—combination chemotherapy consisting of rituximab, cyclophosphamide, vincristine, prednisolone given in 21-day cycles; FOLFOX combination chemotherapy consisting of folic acid, 5-fluorouracil and oxaliplatin; DA 7 + 3—combination chemotherapy consisting of cytarabine given continuously for 7 days and daunorubicin given on 3 consecutive days. [file 13613_2021_867_MOESM1_ESM.docx]

**Table S1**

| **Patient** | **Age** | **Sex** | **Underlying Disease** | **Immunosuppressive medication** |
| --- | --- | --- | --- | --- |
| Graz-CoV-5 | 52 | m | Diffuse large B cell lymphoma | Active therapy with R-CHOP 21 |
| Graz-CoV-13 | 77 | m | Chronic lymphocytic leukemia | Active therapy with rituximab and venetoclax |
| Graz-CoV-19 | 55 | m | Solid organ transplantation | Tacrolimus and mycophenolic acid |
| Graz-CoV-27 | 89 | f | Active multiple myeloma with Secondary immunoglobulin deficiency | Revlimid |
| Graz-CoV-39 | 55 | m | Active multiple myeloma | Day 11 after high dose melphalan 200mg/m² |
| Graz-CoV-44 | 47 | m | Hairy cell leukemia | Active therapy with cladribine (2-CDA) |
| Graz-CoV-45 | 26 | m | Solid organ transplantation | Tacrolimus and mycophenolic acid |
| Graz-CoV-69 | 44 | f | Diffuse large B cell lymphoma | Active therapy with R-CHOP 21 |
| Graz-CoV-75 | 74 | f | Rheumatoid arthritis | Methotrexate 20mg once weekly |
| Graz-CoV-79 | 55 | m | Transformed follicular lymphoma | Active therapy with R-CHOP 21 |
| Graz-CoV-82 | 52 | m | Solid organ transplantation | Tacrolimus and prednisolone 12mg once daily |
| Graz-CoV-85 | 36 | m | Morbus Bruton  (X-linked agammaglobulinemia) |  |
| Graz-CoV-90 | 80 | m | Solid organ transplantation | Everolimus and mycophenolic acid |
| Graz-CoV-99 | 71 | m | Solid organ transplantation | Tacrolimus and cyclosporin A |
| Graz-CoV-100 | 77 | m | Colon cancer | Active therapy with FOLFOX |
| Graz-CoV-105 | 53 | m | Acute myelogenous leukemia | Active therapy with DA 7+3 |
| Graz-CoV-109 | 71 | m | warm antibody autoimmune hemolytic anemia | Active therapy with high dose prednisolone (1.5mg/kg) and Rituximab 375mg/m² once weekly |
| Graz-CoV-119 | 52 | m | Multiple myeloma | Active therapy with daratumumab, carfilzomib and dexamethasone |
