# Supplementary material for: Convalescent plasma therapy and mortality in COVID-19 patients admitted to the ICU: a prospective observational study
Source: Ann Intensive Care. 2021 May 12;11:73. doi: 10.1186/s13613-021-00867-9 (PMC8114671; doi:10.1186/s13613-021-00867-9)
Supplement: Supplementary file 2 — Additional file 2. Supplementary Materials and Methods. [file 13613_2021_867_MOESM2_ESM.docx]

**Supplementary Materials and Methods:**

COVID-19 CVP donation

Patients with a history of SARS-CoV-2 infection confirmed by RT-PCR, which fully recovered from clinical symptoms for at least 2 weeks were considered eligible for plasma donation. (According to the recommendations of the European Commission in the “Guidance on collection, testing, processing, storage, distribution and monitored use of COVID-19 covalescent plasma”)

Female donors with pregnancies in the medical history were screened for HLA -antibodies using the LUMINEX technology. According to the recommendations of the European Commission convalescent plasma donations were allowed, if no antibodies directed against HLA antigens were detectable. Moreover, donors had to fulfill all requirements for blood and plasma donation of the Austrian authority regarding physical examination and history of diseases. Per each donation COVID-19 specific antibodies were tested with the LIAISON SARS-CoV-2 S1/S2 IgG test (DiaSorin, Vienna, Austria). All eligible donors had specific antibodies against the spike glycoproteins S1/S2 of SARS-CoV-2 with a threshold concentration of 30 AU/ml.

Plasmapheresis and quality control

Plasmapheresis was performed using the cell separator Trima Accel^®^ (Terumo BCT, Zaventem, Belgium) and the plasmapheresis system AURORA^®^ (Fresenius Kabi, Graz, Austria). A total of 650 ml convalescent plasma was collected from each donor within 1 hour. Sodium citrate (1:11 - 1:12 ratio) was used as anticoagulant in each apheresis. Blood group typing and red blood cell antibody testing, a screening for infectious agents according to Austrian law was performed. Anti-HIV, anti-HCV, HBsAg, Syphilis, PCR of HIV, HCV, HBV, HAV, HEV and Parvo B19 were tested negative, neopterine was below the limit of 10nmol/L. After transfer to the GMP facility, the CVP was treated with the INTERCEPT Blood System (Cerus E.V., Amersfoort, The Netherlands) a licensed pathogen reduction technology as a second layer of safety and to avoid an additional testing of all plasma donors after a 4-month period of quarantine. This technology enables the blood center to release the CVP immediately after manufacturing.

The plasma donation was divided afterwards into three bags with 200 ml CVP respectively and frozen using a plasma freezer KLF64, (CLST, Poggersdorf, Austria) to a core temperature of -25°C within 45 minutes. The final product has a shelf life of two years.
