# Supplementary material for: Convalescent plasma therapy and mortality in COVID-19 patients admitted to the ICU: a prospective observational study
Source: Ann Intensive Care. 2021 May 12;11:73. doi: 10.1186/s13613-021-00867-9 (PMC8114671; doi:10.1186/s13613-021-00867-9)
Supplement: Supplementary file 4 — Additional file 4: Table S2. Univariable predictors of survival in the cohort (n = 120). BMI: body mass index; first wave: depicts patients treated during the first wave of COVID-19 in Austria between March 2020 and August 2020; mSOFA: modified sequential organ failure assessment, maxPEEP: depicts the maximum applied PEEP to achieve optimal ventilation; IL-6: interleukin 6; CRP-C-reactive protein; hs-TNT: high sensitive troponin T [file 13613_2021_867_MOESM4_ESM.docx]

**Table S2**

| **Variable** |  | **Univariable Hazard Ratio** | **95%CI** | **P** |
| --- | --- | --- | --- | --- |
|  |  |  |  |  |
| **Demographic variables** |  |  |  |  |
| Age (per 5 years increase) |  | 1.30 | 1.15-1.48 | <0.0001 |
| Female Gender |  | 1.44 | 0.82-2.53 | 0.206 |
| BMI (per 5 kg/m² increase) |  | 0.88 | 0.69-1.14 | 0.341 |
| “First wave” |  | 1.49 | 0.82-2.68 | 0.187 |
|  |  |  |  |  |
| **Coexisting conditions** |  |  |  |  |
| Acute respiratory failure grade (inspired by ARDS *Berlin 2012* -classification) |  | 1.15 | 1.03-1.28 | 0.015 |
| Hypertension |  | 0.62 | 0.34-1.15 | 0.127 |
| Diabetes |  | 1.19 | 0.69-2.08 | 0.530 |
| Atrial fibrillation |  | 1.59 | 0.85-2.98 | 0.150 |
| Coronary heart disease |  | 1.20 | 0.66-2.18 | 0.555 |
| Congestive heart failure |  | 2.00 | 1.13-3.54 | 0.018 |
| Peripheral arterial disease |  | 1.82 | 0.99-3.37 | 0.055 |
| Thromboembolic disease |  | 0.89 | 0.40-1.98 | 0.783 |
| Chronic renal failure |  | 3.35 | 1.93-5.81 | <0.0001 |
| Dialysis |  | 2.44 | 1.04-5.75 | 0.041 |
| COPD |  | 1.62 | 0.81-3.22 | 0.173 |
| Asthma |  | 1.21 | 0.52-2.84 | 0.655 |
| Prior cancer in complete remission |  | 1.05 | 0.38-2.91 | 0.931 |
| Active cancer |  | 1.14 | 0.49-2.68 | 0.758 |
| Dementia |  | 2.33 | 0.73-7.48 | 0.155 |
| Prior organ transplantation |  | 2.42 | 0.87-6.73 | 0.091 |
| Immunosuppression |  | 0.92 | 0.41-2.04 | 0.834 |
|  |  |  |  |  |
| **ICU risk stratification** |  |  |  |  |
| mSOFA (per 1 point increase) |  | 1.16 | 1.04-1.29 | 0.008 |
| paO_2_/FiO_2_ (per 10 units increase) |  | 0.90 | 0.85-0.96 | 0.002 |
| maxPEEP (per 1 mmHg increase) |  | 1.13 | 1.02-1.25 | 0.022 |
| Severe ARDS according to *Berlin 2015* -classification |  | 2.61 | 1.31-5.21 | 0.007 |
| Any invasive ventilation |  | 1.39 | 0.79-2.43 | 0.249 |
|  |  |  |  |  |
| **Laboratory values** |  |  |  |  |
| Lactate (per 1 mmol/l increase) |  | 1.23 | 1.13-1.34 | <0.0001 |
| IL-6 (per 50 pg/ml increase) |  | 1.01 | 1.00-1.03 | 0.034 |
| CRP (per 50 mg/l increase) |  | 1.04 | 0.89-1.21 | 0.630 |
| Ferritin (per 1000 ng/ml increase) |  | 1.02 | 0.94-1.11 | 0.616 |
| hs-TnT (per 5 pg/ml increase) |  | 1.00 | 0.99-1.01 | 0.773 |
| D-Dimer (per 1 mg/l increase) |  | 1.00 | 0.96-1.04 | 0.945 |
| SARS-CoV2-Antibody response at ICU admission: Positive |  | 1.07 | 0.57-2.02 | 0.841 |
|  |  |  |  |  |
| **Blood counts** |  |  |  |  |
| Leukocytes [per 1 G/l increase] |  | 1.01 | 0.97-1.05 | 0.633 |
| Neutrophiles [per 1 G/l increase] |  | 1.04 | 0.97-1.06 | 0.485 |
| Lymphocytes [per 1 G/l increase] |  | 0.55 | 0.31-0.96 | 0.036 |
| Thrombocytes [per 100 G/l increase] |  | 0.86 | 0.64-1.16 | 0.332 |
