# Supplementary figures and images for: Convalescent plasma therapy and mortality in COVID-19 patients admitted to the ICU: a prospective observational study
Source: Ann Intensive Care. 2021 May 12;11:73. doi: 10.1186/s13613-021-00867-9 (PMC8114671; doi:10.1186/s13613-021-00867-9)

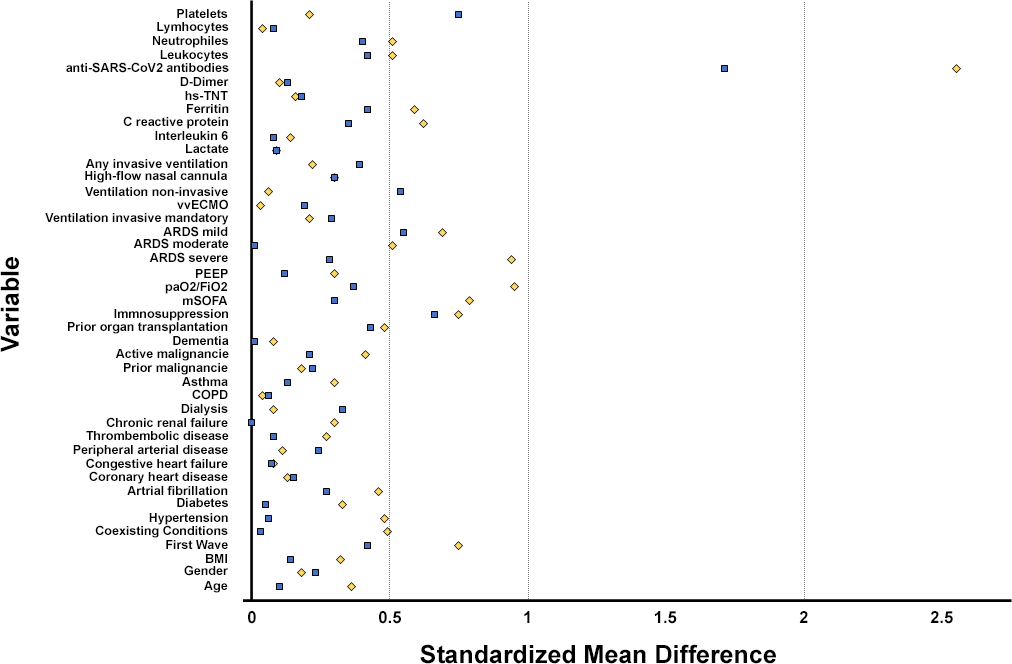

Supplement: Supplementary file 6 — Additional file 6: Figure S3. Standardized mean difference (SMD) plot. Blue squares denote Standardized mean differences (SMDs) in before weighting the inverse of the probability of treatment weight (IPTW). Yellow diamonds denote the SMDs after weighting the IPTW. [file 13613_2021_867_MOESM6_ESM.docx]
