# Supplementary material for: Convalescent plasma therapy and mortality in COVID-19 patients admitted to the ICU: a prospective observational study
Source: Ann Intensive Care. 2021 May 12;11:73. doi: 10.1186/s13613-021-00867-9 (PMC8114671; doi:10.1186/s13613-021-00867-9)
Supplement: Supplementary file 5 [file 13613_2021_867_MOESM3_ESM.docx]

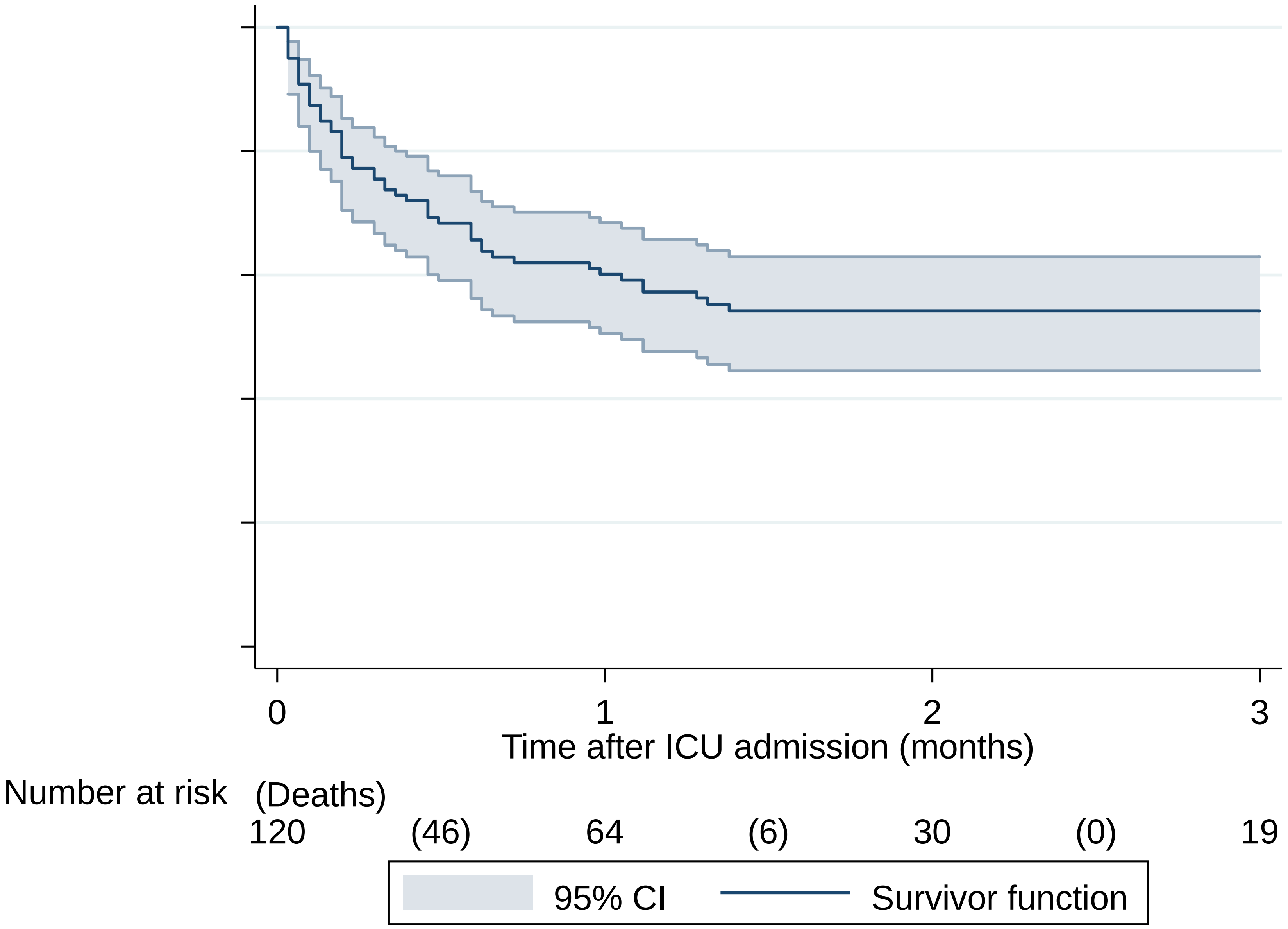
0
1
2
3
Time after ICU admission (months)
Number at risk
(Deaths)
120
(46)
64
(6)
30
(0)
19
95% CI
Survivor function
