# Supplementary material for: Convalescent plasma therapy and mortality in COVID-19 patients admitted to the ICU: a prospective observational study
Source: Ann Intensive Care. 2021 May 12;11:73. doi: 10.1186/s13613-021-00867-9 (PMC8114671; doi:10.1186/s13613-021-00867-9)
Supplement: Supplementary file 6 [file 13613_2021_867_MOESM5_ESM.docx]

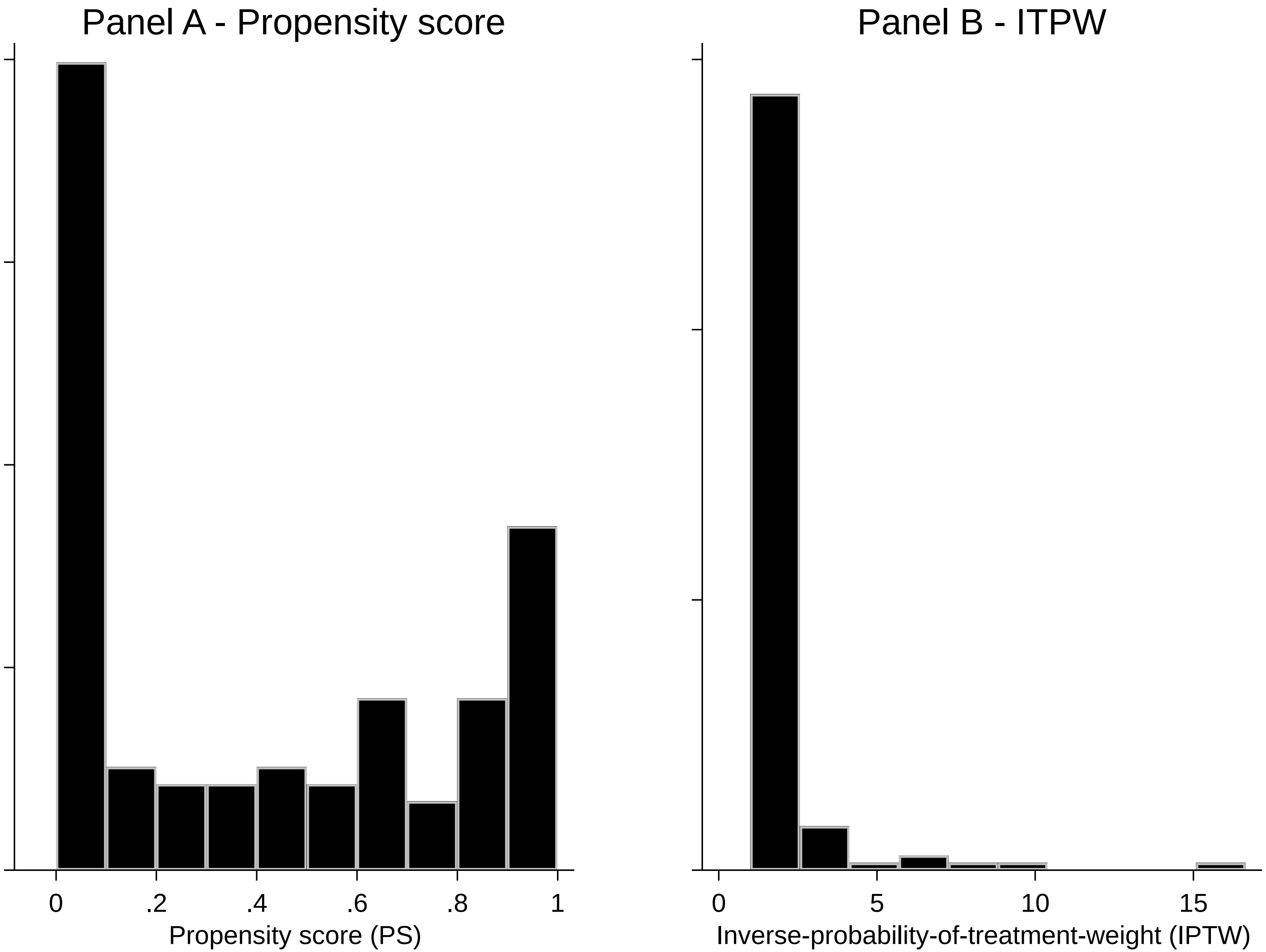
Panel A - Propensity score
0
.2
.4
.6
.8
1
Propensity score (PS)
Panel B - ITPW
0
5
10
15
Inverse-probability-of-treatment-weight (IPTW)
